# Effects of a short period of postural training on postural stability and vestibulospinal reflexes

**DOI:** 10.1371/journal.pone.0287123

**Published:** 2023-06-12

**Authors:** Claudia Grasso, Massimo Barresi, Maria Paola Tramonti Fantozzi, Francesco Lazzerini, Luca Bruschini, Stefano Berrettini, Paolo Andre, Cristina Dolciotti, Vincenzo De Cicco, Davide De Cicco, Paola d’Ascanio, Paolo Orsini, Francesco Montanari, Ugo Faraguna, Diego Manzoni

**Affiliations:** 1 Department of Translational Research and of New Surgical and Medical Technologies, University of Pisa, Pisa, Italy; 2 Department of Surgical, Medical, Molecular Pathology and Critical Cares, University of Pisa, Pisa, Italy; 3 Department of Clinical Science, Intervention and Technology, Karolinska Institutet, Stockholm, Sweden; 4 Department of Medicine, Surgery and Neuroscience, University of Siena, Siena, Italy; 5 Department of Neurosciences, Reproductive and Odontostomatological Sciences, University of Naples “Federico II”, Naples, Italy; 6 Department of Developmental Neuroscience, IRCCS Fondazione Stella Maris, Pisa, Italy; Tokai University, JAPAN

## Abstract

The effects of postural training on postural stability and vestibulospinal reflexes (VSRs) were investigated in normal subjects. A period (23 minutes) of repeated episodes (n = 10, 50 seconds) of unipedal stance elicited a progressive reduction of the area covered by centre of pressure (CoP) displacement, of average CoP displacement along the X and Y axes and of CoP velocity observed in this challenging postural task. All these changes were correlated to each other with the only exception of those in X and Y CoP displacement. Moreover, they were larger in the subjects showing higher initial instability in unipedal stance, suggesting that they were triggered by the modulation of sensory afferents signalling body sway. No changes in bipedal stance occurred soon and 1 hour after this period of postural training, while a reduction of CoP displacement was apparent after 24 hours, possibly due to a beneficial effect of overnight sleep on postural learning. The same period of postural training also reduced the CoP displacement elicited by electrical vestibular stimulation (EVS) along the X axis up to 24 hours following the training end. No significant changes in postural parameters of bipedal stance and VSRs could be observed in control experiments where subjects were tested at identical time points without performing the postural training. Therefore, postural training led to a stricter control of CoP displacement, possibly acting through the cerebellum by enhancing feedforward mechanisms of postural stability and by depressing the VSR, the most important reflex mechanism involved in balance maintenance under challenging conditions.

## Introduction

Similarly to voluntary movements, postural control may undergo learning processes, increasing the ability of maintaining balance [1]. Specific exercises challenging balance and sport activities requiring balance abilities [1–4] are most powerful in improving postural control, but also non-specific exercises can be effective [1].

So far, the effects of postural training have been mostly investigated over rather long time lapses (from 5 weeks to 3 months). Experiments have been conducted in people of different gender, age and ability, resorting to a wide range of experimental paradigms, rather heterogeneous in terms of duration of training periods, frequency/duration of individual training sessions, as well as total training time per week [5, 6].

Postural training increased the ability to maintain balance under challenging conditions, such as tandem [7] and unipedal stance [7–11], leading to a reduction of trunk acceleration [12] and of the area covered by the Centre of Pressure (CoP) [1, 13, 14].

There is evidence that postural improvement elicited by balance training is specific. For instance, expert gymnasts, who usually practise with their eyes open, show a superior balance than sedentary people only when vision is allowed under challenging postures [15]. Apparently, in this population, the acquired postural ability does not transfer from a posture to another [16]. Similarly, stability in normal, bipedal stance is not improved when a subject acquires the skill to maintain balance on a slackline [1]. Other studies, however, report that postural training performed by standing in unipedal stance or on unstable surfaces may reduce the area of CoP motion observed in the normal bipedal stance [13, 14].

Only few studies have so far addressed the short-term effects of balance training on postural stability: there is evidence that practising on an unstable board for 6–16 minutes increases the time a subject can stand without falling in this unstable condition, from about 1.5 to 4 seconds [17]. Moreover, the ability of balancing on unstable support in unipedal stance is increased following 10–30 minutes of training [18]. None of these studies, however, have addressed the progressive evolution of postural stability by analysing the characteristics of CoP motion during short-term training in a challenging posture such as unipedal stance.

We may expect that an improvement in postural performance is associated with modifications in the visual, vestibular, proprioceptive and somatosensory reflexes [19, 20] which contribute to postural stability. Somatosensory [21, 22] and vestibular [23] reflexes involve both short and long latency pathways and can be modulated by the cerebral cortex, the cerebellum and the basal ganglia [21].

There is evidence that long periods of postural training may decrease the amplitude of the soleus stretch [24] and of the H-reflex [25–28]. The depression of the H-reflex could be specific for the training posture/task [26, 28] or could otherwise generalise to normal bipedal stance on firm support [25]. In other long-term studies, however, postural training did not elicit H-reflex depression at all, either in the untrained or in the trained condition [29–31]. Finally, an increase in H-reflex amplitude has been reported in subjects tested following 6 weeks of combined postural and strength training [32], as well as following a 12-week period of Tai Chi Yang training [33, 34], implying wide stance width and slow speed of movement during practice, leading to an improvement of lower limbs muscle strength by imposing constant knee flexion.

Only few studies addressed the effects of short periods (10–20 minutes) of postural training, showing a decrease in H-reflex amplitude evaluated while sitting, standing in unipedal stance [17] or standing on an unstable surface [35].

As to the effect of postural training on the short and long latency electromyographic (EMG) responses evoked by the translation of the support surface, no modifications have been shown following four weeks of balance training [36, 37]. At variance, responses to rotation of the support surface in the sagittal plane, which further destabilise the subjects [38] are decreased following three days of training [39]. Finally, 13 days of balance training reduce the latency and increase the amplitude of the late component of the EMG responses to gait perturbation [40].

Long-term postural training has been shown to also modify the cortical control on postural reflexes, as indicated by post-training reduction of the cortical elicited facilitation of the H-reflex is with respect to control [30, 36]; interestingly, this phenomenon is associated to an enhancement of intracortical inhibition [41].

No study has so far addressed the modifications elicited by balance training on postural changes evoked by stimulation of the labyrinth, which is the sensory system in charge of maintaining balance under challenging conditions, such as when standing on unstable surfaces or when assuming tandem and unipedal stance [42–44].

The aims of the present investigation were:

1. to study the progressive evolution of postural stability in unipedal stance by analysing the characteristics of CoP motion during and after a short-term training in this posture,
2. to verify whether this training also modifies the reflex responses arising from labyrinthine stimulation.

## Materials and methods

### Subjects

Twenty-one healthy volunteers (9 women and 12 men, 31.6±7.7, mean age ± SD) free from cardiovascular, neurological and orthopaedic problems were enrolled in the experiment. The state of health of participants was assessed by a preliminary talk with the researchers of our team who asked whether they were suffering of cardiovascular, respiratory, metabolic, neurological, vestibular, psychiatric and orthopaedic symptoms. Only subjects who did report absence of these medical problems were enrolled in the study. Eighteen of them (9 women and 9 men) were right handed, while 3 left handed. The study was approved by the Committee on Bioethics of the University of Pisa (Protocol Number: 28/2022) and the subjects signed a written informed consent.

### Experimental design

As shown in Table 1, the experimental design included four different sessions (2 training and 2 control sessions).

**Table 1:** Experimental design and time course.

		Experimental Time Course				
		Baseline	Postural Training /Rest Period (~23 min)	Time 0h	Time 1h	Time 24h
**A**	**Training-Stance Session** **N = 11**	Bipedal Stance OE-HS CE-HS OE-SS CE-SS	Unipedal Stance OE-HS	Bipedal Stance OE-HS CE-HS OE-SS CE-SS	Bipedal Stance OE-HS CE-HS OE-SS CE-SS	Bipedal Stance OE-HS CE-HS OE-SS CE-SS
**B**	**Control-Stance Session** **N = 8**	Bipedal Stance OE-HS CE-HS OE-SS CE-SS	Rest	Bipedal Stance OE-HS CE-HS OE-SS CE-SS	Bipedal Stance OE-HS CE-HS OE-SS CE-SS	Bipedal Stance OE-HS CE-HS OE-SS CE-SS
**C**	**Training-EVS Session** **N = 13**	Bipedal Stance VSR	Unipedal Stance OE-HS	Bipedal Stance VSR	Bipedal Stance VSR	Bipedal Stance VSR
**D**	**Control-EVS Session** **N = 10**	Bipedal Stance VSR	Rest	Bipedal Stance VSR	Bipedal Stance VSR	Bipedal Stance VSR

The temporal sequence of stabilometric and neurophysiological recordings has been illustrated for the different experimental sessions. EVS: Electrical Vestibular Stimulation. OE: Open Eyes. CE: Closed Eyes. HS: Hard Support. SS: Soft Support. VSR: Vestibulo-Spinal Reflex. N refers to the numbers of subjects involved in each experimental session.

Subjects were invited to avoid any excess in alcohol, coffee and smoking on the experimental day and the preceding one. Only six of the subjects successfully completed all four sessions, occurring at least three months apart. The training-stance sessions (Table 1A) began with the measurement of the Centre of Pressure (CoP) by a stabilometric platform, during barefoot bipedal stance, each lasting 50 seconds. Recordings were taken with eyes open (OE) and closed (CE), on hard (HS) and soft support (SS), according to the following sequence: OE-HS, CE-HS, OE-SS, CE-SS. Subjects were allowed to sit for 60–120 seconds between HS and SS recordings. During OE recordings, subjects were asked to fixate a black spot (2 cm of diameter), at a distance of 90 cm, at the eyes level, while during CE recordings they were invited to keep the same gaze position. Following these initial bipedal stance recordings, after 60–120 seconds of rest, subjects underwent a postural training period, lasting about 23 minutes. The training protocol consisted of 10 successive periods of unipedal stance, in OE-HS conditions, performed at regular intervals (90 seconds). Subjects were allowed to sit between a training period and the following one. During each training period the CoP displacement was recorded. Subjects were invited to choose their dominant leg for standing. 90 seconds after the training (Time 0h), 1 hour (Time 1h) and 24 hours (Time 24h) later, the subjects repeated the whole sequence of CoP displacement recordings in bipedal stance.

In the control-stance sessions (Table 1B), participants were submitted to all the sequence of bipedal stance CoP measurements performed during the training session, but the 23 minutes postural training period was substituted by a resting period of the same duration.

In the training-EVS session (Table 1C) participants underwent, on hard support and closed eyes, electrical bipolar stimulation of the labyrinth (Electrical Vestibular Stimulation, EVS), which gave rise to stimulus-locked CoP displacements and leg postural muscles EMG responses (Vestibulo Spinal Reflexes, VSRs). During EVS the feet were always touching at the heel and positioned at an angle of 35°, bisected by the medial sagittal plane. EVS was delivered while participants were asked to keep their head in three different positions: forward (HF), leftward (HL), rightward (HR). In each position the stimulation lasted for about two minutes. Subjects were asked to maintain HL and HR orientations avoiding any effort. The angle of head-to-body rotation was evaluated before and after each recording with the help of a 180° circular sector with a 30 cm radius placed on the subject’s shoulders. Moreover, the stability of head position was visually controlled by an experimenter during the entire recording session. Trunk displacements associated with head rotation were corrected according to the instruction of the experimenters. Subjects were allowed to sit with open eyes for about 60 seconds between consecutive trials. Then, each subject underwent the same postural training (with simultaneous recording of CoP displacement) as the one performed in the training-stance session.

The whole sequence of EVS elicited in HF, HR and HL was repeated soon after, 1 hour and 24 hours following the postural training.

In the control-EVS session (Table 1D), participants were submitted to EVS at the same times as in the training/EVS session, but instead of performing the postural training they were invited to rest for a corresponding time span.

### Labyrinthine stimulation

In the present study EVS was performed by using trains of pulses (1 msec pulse duration, train duration 300 msec), applied through surface electrodes placed over the mastoid bones (cathode always placed on the right side). The interpulse interval was fixed at 40 μsec, which produced a *quas*i-constant current [45]. These stimuli were delivered by an isolated, constant-current stimulator (Digitimer model DS7A) driven by a PC. The intensity of the current was checked at the end of the stimulation by measuring the voltage drop induced by the current across a 1 KΩ resistor. The stimulus intensity was 1.2 times the lower stimulus intensity (threshold) inducing a perception of body sway without autonomic symptoms and/or visual flash hallucinations. The applied current intensity values ranged from 0.4 to 2.0 mA. To elicit unpredictable stimuli, the PC generated a sequence of train pulses with a randomly varying interval at the average frequency of 0.35 Hz. Typically, 30–45 CoP and EMG responses to vestibular stimulation were recorded for each of the three head positions.

### CoP and EMG recordings

The position of the CoP was recorded through a stabilometric platform (Dune 2000; Dune S.A.R.L., Negenheim, France), endowed with three force sensors disposed at the apices of an equilateral triangle having one of the sides parallel to the platform posterior edge. CoP coordinates were centred within the centre of the triangle, about 30–40 cm in front of the habitual CoP position. Average CoP X and Y coordinates, Standard Deviation (SD) of CoP position on both X and Y axes (corresponding to the average CoP oscillation with respect to the mean position), average CoP velocity, 95% ellipse area (the area of the ellipse containing 95% of the CoP positions) and LFS (cumulative length of CoP displacement/95% ellipse), evaluated over the recording time (50 seconds) were automatically calculated by the platform software.

To evaluate the parameters of the stimulus-locked CoP motion, the original signals from the force sensors were separately acquired and analysed through a Lab-view software prepared ad hoc (sampling rate 2KHz), which calculated the time course of CoP’s X and Y coordinates.

EMG recordings were taken from the medial (GM) and lateral (GL) gastrocnemius of both sides. EMG signals were amplified and filtered through a Lace amplifier (amplification 10000, high-pass frequency 0.5 Hz, low-pass frequency 5000 Hz). A further step of filtering was performed off-line on the acquired digital data by a custom-made Labview software: the low frequency cut-off was set to 5–10 Hz and the high cut-off frequency to 800 Hz when high frequency artefacts due to the stimulation train occurrence. Finally, EMG traces were full-wave rectified before evaluation of time-locked responses to EVS.

### Time-locked CoP and EMG responses to EVS

**Fig 1:**
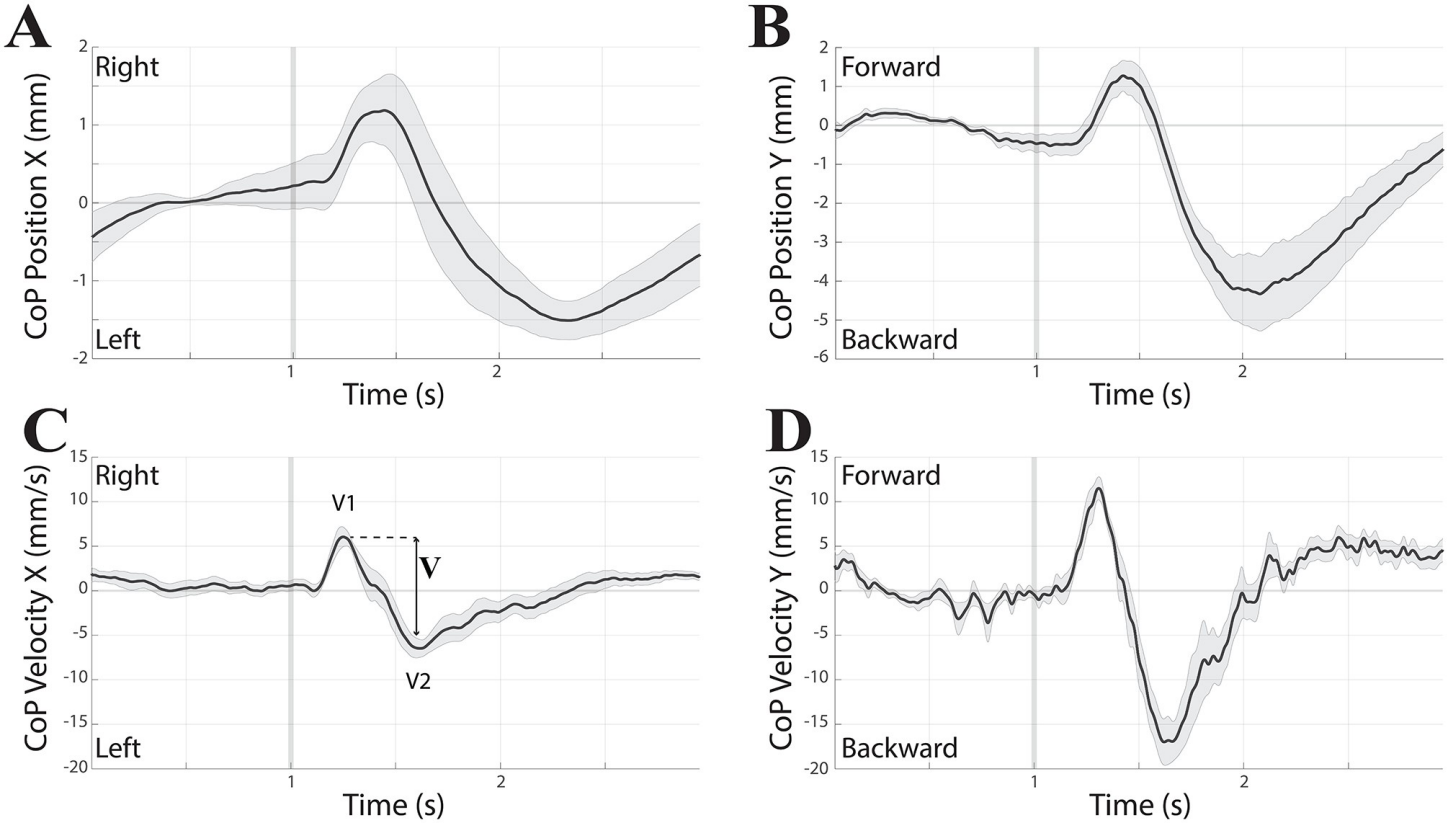
Effects of EVS on CoP position and velocity. Grand average of CoP position (A, B) and velocity (C, D) changes elicited by EVS delivered in HL position. Modifications elicited along the X and Y axes are displayed in A/C and B/D, respectively. All the subjects analysed over a 3 seconds time frame have been included (n = 11). In each panel the grey area encompassing the average trace represents SE, while the vertical grey bar depicts the stimulus onset.

A Lab-view, custom-made software was used to obtain stimulus-locked CoP and EMG responses. For this purpose, CoP coordinates were referred to the average of the X and Y CoP values relative to the pre-stimulus period. 30–45 successive sweeps were averaged over epochs of 3 seconds (pre-stimulus: 1 second; post-stimulus: 2 seconds). Rarely (overall x out of y trials), for unintentional technical reasons, the recorded data covered only a 1.5 seconds time span (pre-stimulus: 0.5 second; post-stimulus: 1 second). The obtained CoP traces were submitted to a smoothing procedure in which a given point (at the N^th^ position in the trace) was substituted by the average of all the points (101) included between N+50 and N-50. This procedure obliged to discard the first 50 and the last 50 points of each trace but did not modify the response time course. Examples of EVS-elicited responses of CoP position along X and Y axes are shown in Fig 1A/1B, respectively. In Fig 1, positive and negative values of the X component (Fig 1A) represent right and left displacements, respectively, while positive and negative values of the Y component (Fig 1B) correspond to forward and backward displacements. CoP responses were biphasic. In this representative case the initial displacement took place forward and rightward and the second one was in the opposite directions. Fig 1C and 1D show the instantaneous rate of change (CoP Velocity), digitally computed and smoothed (101 points), as the first derivative of the data depicted in Fig 1A and 11B, respectively.

Yet, positive and negative values of the X velocity component (Fig 1C) represent right/left directions of movement, while positive and negative values of the Y component (Fig 1D) refer to forward and backward directions of movement. Both components showed biphasic changes in opposite directions (rightward/leftward and forward/backward), the corresponding peaks being indicated as V1 and V2. The CoP is continuously modified by the changes in feet push onto the platform and in spatial position of the centre of mass, both of them being affected by VSR. On account of this, both components are reliable indicators of the VSR strength. Although the initial displacement of CoP velocity trace is related to feet push, the exact contribution of the two components to CoP velocity trajectory cannot be disentangled. For this reason, in addition to the V1 and V2 components, the peak to peak excursion (V = V2-V1) of CoP velocity traces was evaluated as a measure of the amplitude of CoP velocity response to vestibular stimulation.

The V values obtained for the X components were always negative (final CoP velocity to the left), regardless of head position. As to the Y components, V values were positive in HR (final CoP velocity directed forward) and negative (final CoP velocity directed backward) in HL, while nihil in HF.

**Fig 2:**
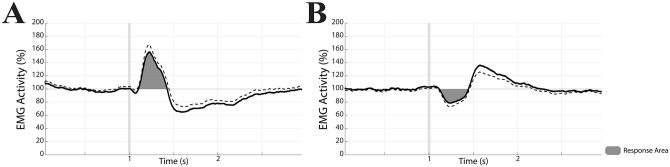
Effects of EVS on EMG activity. Grand average of the EMG response of the right GM to EVS in HL (A) and HR (B) position. All the subjects analysed over a 3 seconds time frame are included (HL: n = 10, HR: n = 11). The surface of the grey area underlying the peak activity (response area) was computed as a measure of the EMG evoked-response strength. In each panel the dotted line represents SE, while the vertical grey bar is the stimulus onset. Data are expressed as a percentage of the average value evaluated over the pre-stimulus period.

The rectified EMG traces were submitted to 201 points smoothing to decrease the high frequency noise component and normalised as percentage of the pre-stimulus baseline values. Post-stimulus increases in EMG activity with respect to baseline corresponded to muscle contraction, while reduced values indicated muscle relaxation. As shown in Fig 2, EMG responses were in general biphasic, with an early peak consisting either in contraction (Fig 2A) or relaxation (Fig 2B) followed by a second component of opposite sign. As a first step, we evaluated, by visual inspection, the points where the EMG traces raised above (response latency) and went back to the baseline values (response end) following the stimulation. As shown in Fig 2, the early responses were quantified, whatever their nature (contraction or relaxation), as the area under the first component response curve (response area).

### Statistical analysis

In the Training-stance and in the Training-EVS sessions, stabilometric unipedal stance parameters sampled 10 times every 2.3 minutes, were averaged in pairs of two, so to obtain 5 time points, each encompassing a time window of 4.6 minutes. For each session and variable, changes induced by postural training were analysed by a 5 Time repeated measures ANOVA, with LSD as post-hoc test.

Moreover, all data were expressed as a percentage of the value obtained by averaging the 5 time points of each individual subject. Data from different subjects were pooled together and correlated with time. The latter analysis was performed by using linear, logarithmic, inverse and exponential models.

In all the four sessions, possible modifications observed in bipedal stance parameters soon after postural training/rest periods, at 1 and 24 hours with respect to baseline (BSL) were analysed by a 4 Time (BSL, 0h, 1h, 24h) x 2 Eye (OE, CE) x 2 Support (HS, SS) repeated measures ANOVA. As to stimulus-locked CoP velocity responses to EVS, a 4 Time (BSL, 0h, 1h, 24h) x 3 Head position (HF, HR, HL) repeated measures ANOVA was applied to V values. In this instance, zero amplitude values indicated lack of CoP responses to labyrinthine stimulation. In this analysis, X and Y components of responses were analysed separately. Since V had opposite sign in HR (positive) with respect to HL (negative), ANOVA was applied to the corresponding absolute values. The amplitude of stimulus-locked EMG responses were analysed by a 4 Time (BSL, 0 h, 1h, 24h) repeated measures ANOVA, separately applied to the different head positions and muscles. All the statistical computations were performed by a SPSS software package. When data distribution did not respect the sphericity assumption, p values were corrected as appropriate.

## Results

### Training-induced changes in unipedal stance: Training-stance session

**Fig 3:**
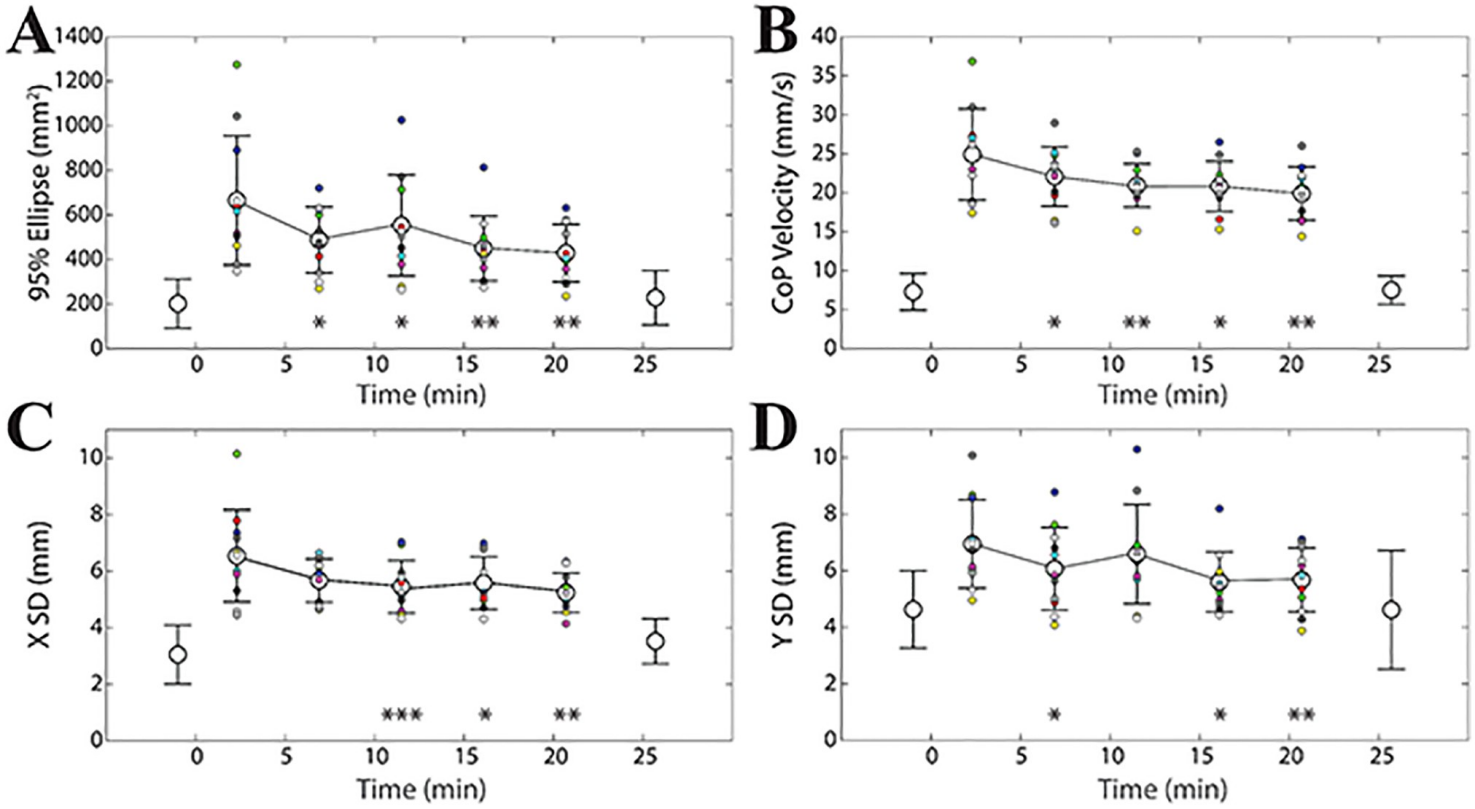
Training-induced changes in unipedal stance. Mean±SD values of 95% ellipse (A), CoP velocity (B), X SD (C) and Y SD (D) evaluated in unipedal stance at different time points during postural training. Data plotted in light grey, before and after the training interval, represent average values obtained in bipedal stance on hard support, eyes open. Asterisks indicate significant differences with respect to the first training time interval (0–4.6 minutes). *: p<0.05; **: p<0.02; ***: p<0.01. Single subject data relative to the training period can be identified on the basis of a colour code.

Analysis of the data collected during unipedal stance in the training-stance session revealed significant time effects for 95% ellipse (F(4,40) = 6.143, p = 0.001), CoP velocity (F(4,40) = 7.39, p = 0.006), X SD (F(4,40) = 5.853, p = 0.006) and Y SD (F(4,40) = 3.969, p = 0.007). Post-hoc analysis indicated that, in general, 95% ellipse, CoP velocity, X SD and Y SD showed significant decreases across training with respect to the first time interval, encompassing the initial 4.6 minutes. These data are summarized in Fig 3, where the initial and final values obtained in bipedal stance on hard support and open eyes were also reported. No significant Time effect could be observed for LFS.

Another evaluation of the training effect was performed by correlation analysis. For this purpose, in each subject, all parameters were normalised (as %) with respect to the average value obtained during the training period. Data from different subjects were pooled together. Except for LFS, all parameters appeared to be negatively correlated with time. All the tested models were well fitting the experimental data: average values of correlation coefficients evaluated across parameters (except LFS) corresponded to 0.534, 0.575, 0.574 and 0.538 for the linear, logarithmic, inverse and exponential model, respectively. In particular, the correlation coefficients (R) of the logarithmic models corresponded to 0.610 (95% ellipse, Y = -18.060lnX+139.790, p<0.0005), 0.589 (X SD, Y = -9.002lnX+119.833, p<0.0005), 0.450 (Y SD, Y = -8.534lnX+118.801, p = 0.001) and 0.652 (CoP velocity, Y = -9.735lnX+121.448, p<0.0005).

Training-induced changes in the different variables were correlated to each other. This analysis was based on the last two points of the training session and the degree of such changes was evaluated as the difference relative to the initial time point (2.3 minutes, see Fig 3). Changes in 95% ellipse strongly covaried with those in X SD (R = 0.76, p<0.0005, Y = 138.72X-44.33), Y SD (R = 0.79, p<0.0005, Y = 129.72X-65.82) and CoP velocity changes (R = 0.77, p<0.0005, Y = 39.27X-33.93). CoP velocity changes were significantly correlated with both X SD (R = 0.835, Y = 3.014X-0.648, p<0.0005) and Y SD (R = 0.474, Y = 1.569X–1.978, p<0.0005) changes, while the correlation between X and Y SD changes did not reach significance level (R = 0.26, p = 0.084).

**Fig 4:**
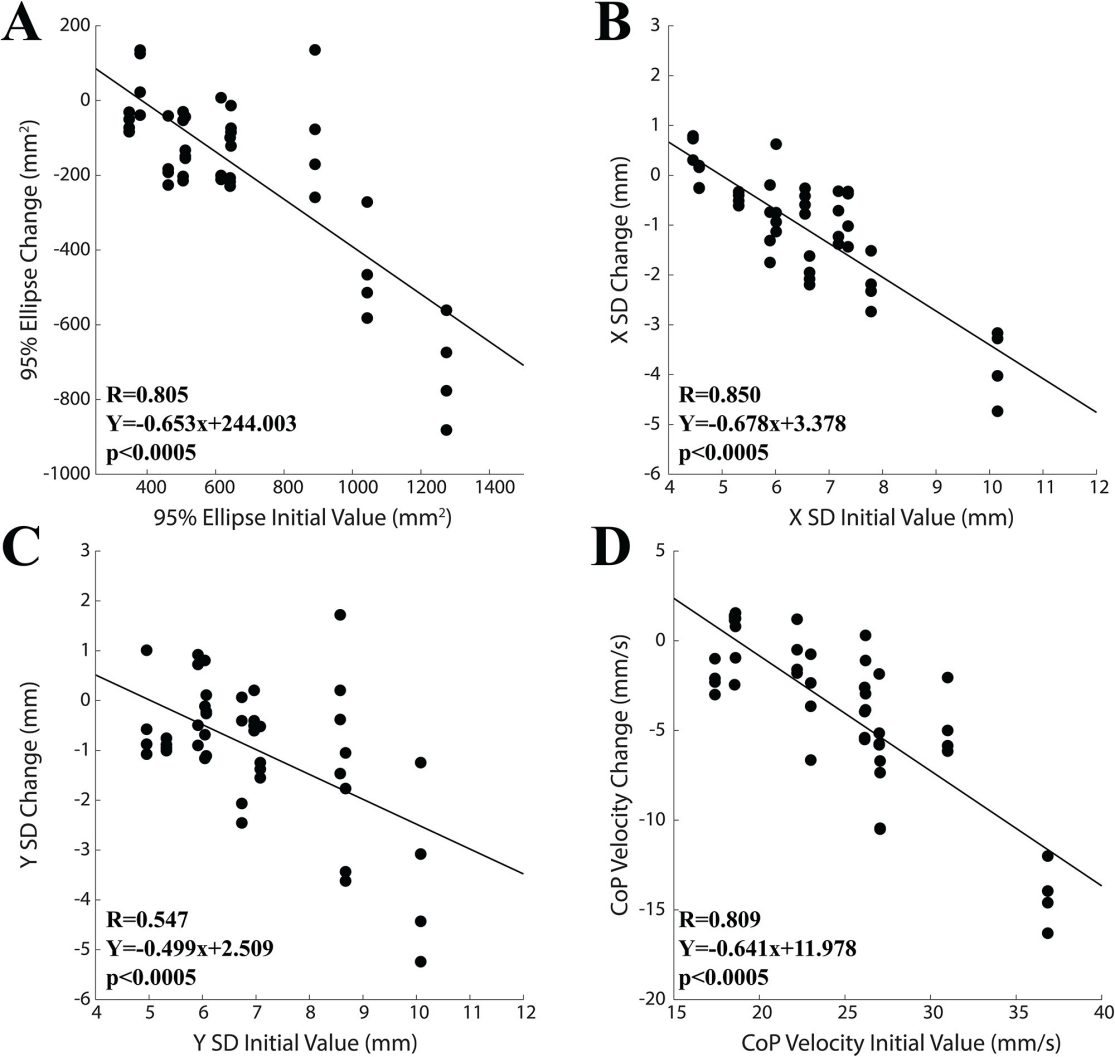
Correlation of training-induced changes in postural variables with their initial training interval values. Data relative to the second (6.9 minutes), third (11.5 minutes), fourth (16.1 minutes) and fifth (20.7 minutes) time points of the training interval have been reported. A: 95% ellipse. B: CoP velocity. C: X SD. D: Y SD. In all the scatterplots data in ordinate correspond to the difference between the variable value at a given time point and the initial time point value (2.3 minutes). The lines are regression lines evaluated for all the plotted points.

It was observed that the training-induced changes in the recorded variables, evaluated as difference with respect to the initial training interval value (2.3 minutes, see Fig 3), were significantly correlated with their initial amplitude: larger initial values corresponded to larger training-induced drops. This held true for all the variables at the last two (16.1 and 20.7 minutes, see Fig 3) time points and for 95% ellipse, X SD and CoP velocity also at the second and third time points (6.9 and 11.9 minutes, see Fig 3). These data are summarized in Fig 4, where changes obtained at 6.9, 11.9, 16.1 and 20.7 minutes for each subject have been cumulatively plotted with respect to the initial values (95% ellipse: R = 0.805, Y = -0.653X+244.00, p<0.0005; X SD: R = 0.850, Y = -0.678X+3.378, p<0.0005; Y SD: R = 0.547, Y = -0.499X+2.51, p<0.0005; CoP velocity: R = 0.809, Y = -0.641X+11.978, p<0.0005).

### Changes in bipedal stance induced by unipedal postural training

Stabilometric data recorded in bipedal stance in the training-stance session were recorded at four time points in two different conditions of Support (soft, hard) and Eye (open, closed), so that they were initially analysed by a three-way ANOVA model (4 Time (BSL, 0h, 1h, 24h) x 2 Support (HS, SS) x 2 Eye (OE, CE) repeated measures ANOVA). A Time effect was observed for 95% ellipse (F(3,30) = 3.13, p = 0.040) (Table 2A) due to a 24h value significantly or quasi significantly lower than all the preceding data points (BSL: p = 0.026, Time 0h: p = 0.051, Time 1h: p = 0.056). In the control-stance session, no significant Time effect or interactions were observed.

**Table 2:** Average±SE values of 95% ellipse.

		Baseline	Time 0h	Time 1h	Time 24h
A	**Training-stance Session**	403.9±59.0	373.3±62.8	365.2±53.7	292.1±37.1
			NS	NS	*
	**Control-stance Session**	392.1±70.1	392.3±60.0	391.1±40.2	385.2±72.0
			NS	NS	NS
B	**Training-stance Session CE condition**	472.5±65.9	428.6±68.3	417.7±62.7	321.3±32.2
			NS	NS	*
	**Training-stance Session SS condition**	570.4±93.3	507.2±108.3	535.3±94.2	357.5±25.2
			NS	NS	*

Data have been recorded at the different times in training-stance and control-stance sessions. Asterisks indicate that the difference between the corresponding time point value (Time 24h) and the initial one (Baseline) was statistically significant (p<0.05).

Since interactions between factors showed eta^2^ values lower than 0.2, we also run two-way ANOVA models. Indeed, a 2 Support (HS, SS) x 4 Time (BSL, 0h, 1h, 24h) repeated measures ANOVA and a 2 Eye (OE, CE) x 4 Time (BSL, 0h, 1h, 24h) repeated measures ANOVA were run separately for the OE and CE conditions and for the HS and SS conditions, respectively. These analyses revealed a significant Time effect for 95% ellipse only under CE (F(3,30) = 3.458, p = 0.029) and SS conditions (F(3,30) = 2.948, p = 0.049). This effect is decomposed in Table 2B. The 24h value of 95% ellipse was significantly lower than both baseline (p = 0.01) and Time 0h (p = 0.048) under the CE condition. With SS, only the difference between baseline and Time 24h reached the significance level (p = 0.034). Moreover a significant Time effect could be also observed for Y SD (F(3,30) = 5.364, p = 0.004) with CE, being the 24h value (5.23±1.23, mm) significantly lower than both baseline (6.46±1.65, mm, p = 0.002) and 0h (5.82±1.59, p = 0.046) values. No significant effects and interactions could be found when these ANOVA models were applied within the control-stance session.

### Postural training and vestibular stimulation

**Fig 5:**
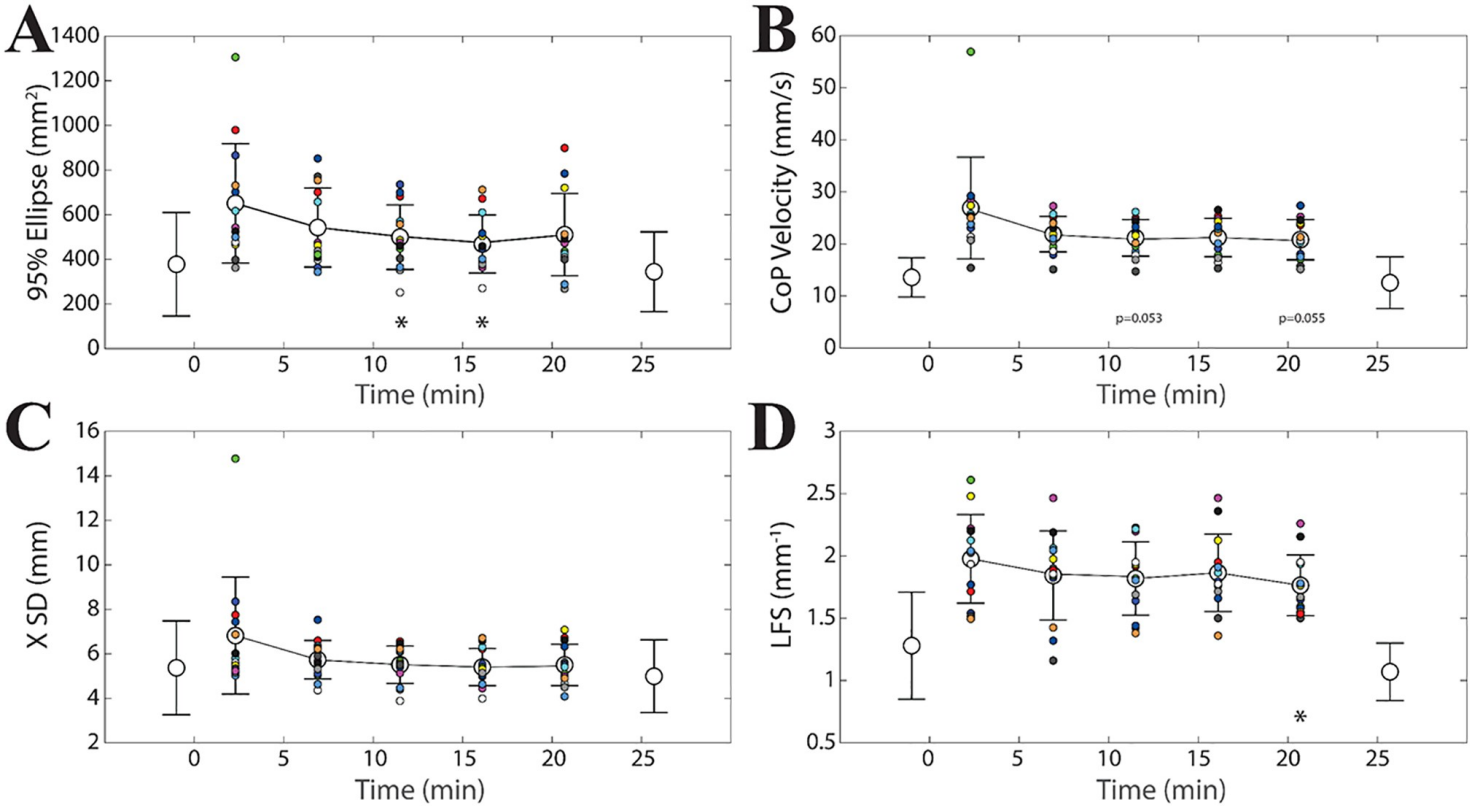
Training-induced changes in unipedal stance observed following EVS. Mean±SD values of 95% ellipse (A), CoP velocity (B), X SD (C) and LFS (D) evaluated in unipedal stance at different time points during postural training performed following EVS. Data plotted in light grey, before and after the training interval, represent average values obtained in bipedal stance on hard support, eyes open. Asterisks indicate significant differences with respect to the first training time interval (0–4.6 minutes). *: p<0.05; **: p<0.02; ***: p<0.01. Single subject data relative to the training period can be identified on the basis of a colour code.

With respect to the training-stance session, following EVS, significant/quasi-significant Time effects could be observed only for 95% ellipse (F(4,48) = 2.974, p = 0.028) and CoP velocity (F(4,48) = 3.889, p = 0.06), when recorded in unipedal stance. In the training-EVS session, however, a significant Time effect could be observed also for LFS (F(4,48) = 2.784, p = 0.037). All variables decreased during training with respect to the initial training interval values: average and single subjected data can be found in Fig 5 for 95% ellipse, X SD, CoP velocity and LFS.

The regression analysis of postural variables with respect to time revealed, however, a significant decrease in all the parameters (LFS included), whatever the model applied. Average R values evaluated across parameters corresponded to 0.353, 0.409, 0.428 and 0.361 for the linear, logarithmic, inverse and exponential models, respectively. In particular, R values relative to the logarithmic model corresponded to 0.428 (95% ellipse, Y = -13.802lnX+130.408, p<0.0005), 0.436 (X SD, Y = -10.022lnX+122.080, p<0.0005), 0.318 (Y SD, Y = -10.420lnX+122.957, p = 0.010), 0.493 (CoP velocity, Y = -11.475lnX+125.280, p<0.0005) and 0.372 (LFS, Y = -4.187lnX+109.223, p = 0.002).

Consistently with the training-stance session, the changes in the different parameters were always negatively correlated with the initial values (95% ellipse: R = 0.818, Y = -0.833X +397.00, p<0.0005; CoP velocity: R = -0.933, Y = -0.957X+20.11, p<0.0005; X SD: R = 0.946, Y = -0.964X+5.292, p<0.0005, Y SD: R = 0.859, Y = -0.815X+5.259, p<0.0005; LFS: R = 0.507, Y = -0.405X+0.678, p<0.0005). As observed in the training-stance session (without EVS), the changes in the different parameters were correlated to each other (Table 3). In the training-EVS session, however, unlike the training-group, a correlation was found also between changes in Y SD and X SD.

**Table 3:** Training-EVS session.

**95% Ellipse**	1				
**CoP Velocity**	R = 0.846, p<0.0005	1			
**X SD**	R = 0.920, p<0.0005	R = 0.933, p<0.0005	1		
**Y SD**	R = 0.891, p<0.0005	R = 0.967, p<0.0005	R = 0.911, p<0.0005	1	
**LFS**	R = 0.268, p = 0.055	R = 0.711, p<0.0005	R = 0.494, p<0.0005	R = 0.576, p<0.0005	1
	**95% Ellipse**	**CoP Velocity**	**X SD**	**Y SD**	**LFS**

Correlation coefficients of the regression analysis performed between changes in the different parameters of unipedal stance observed at the end of the training period.

### Training-induced changes in VSRs: Kinematics

**Fig 6:**
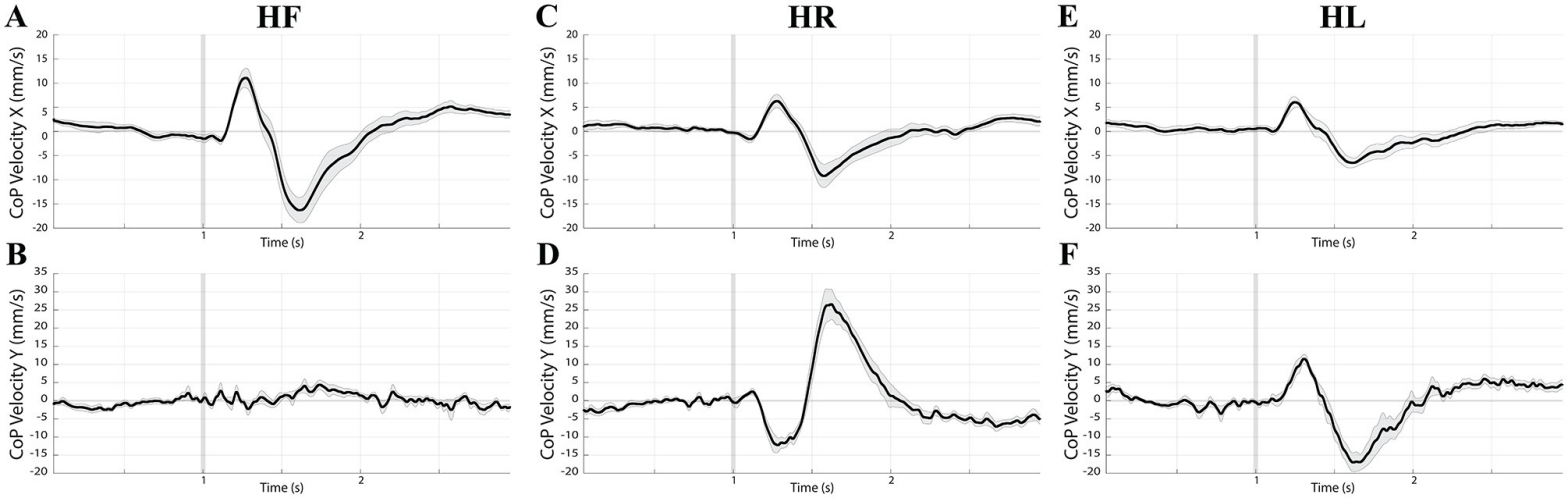
Head position effects on EVS-elicited changes in CoP velocity. Grand average of baseline CoP velocity changes elicited by EVS delivered in HF (A, B, n = 10), HR (C, D, n = 12) and HL (E, F, n = 11) position. Panels A, C, E and B, D, F, represent modifications elicited along the X and Y axes, respectively. All the subjects analysed over a 3 seconds time frame have been included. In each panel the grey area encompassing the average trace represents SE, while the vertical grey bar is the stimulus onset.

The changes in CoP velocity typically induced by EVS along X and Y axes were biphasic, with the two components (V1 and V2) occurring in opposite directions. This can be appreciated in Fig 6, where baseline data have been shown. The peak to peak changes along the X axis were larger in the HF position with respect to both HR (p = 0.002) and HL (p<0.0005), while the opposite held true for Y displacements, which were generally absent in HF. Along the X axis, V1 was always oriented to the cathode side (right side, positive values in Fig 6), while V2 to the anode side (left side, negative values). Along the Y axis V1 was directed backward (negative values) and forward (positive values) when the head was in HR and in HL, respectively. The opposite held true for V2.

The peak to peak change along the Y axis was larger than that along the X axis in both HR (p<0.0005) and HL (p = 0.002) position. The opposite held true in HF (p<0.0005).

Data along both axes were separately analysed to verify possible training-induced changes in VSRs. A 4 Time (BSL, 0h, 1h, 24h) x 3 Head (HF, HR, HL) and 4 Time (BSL, 0h, 1h, 24h) x 2 Head (HR, HL) repeated measures ANOVA were used for analysing the X and Y components of CoP velocity (V), respectively. In the latter analysis, both HR and HL data were taken with their absolute values.

It must be pointed out that the angle of head-to-body rotation observed in both HR and HL was remarkably stable across the whole session. HR and HL values obtained at the different time points were correlated with each other, with high coefficients of correlation (0.974–0.993) and slopes values close to 1 (0.937–1.024). In HR, the average angles observed at the beginning of the baseline and 24h time points were 45.6±8.9, SD, ° and 43.7±8.9, SD, °, respectively. In HL, baseline and 24h values corresponded to 133.4±6.6, SD, ° and 135.1±9.4, SD, °.

**Fig 7:**
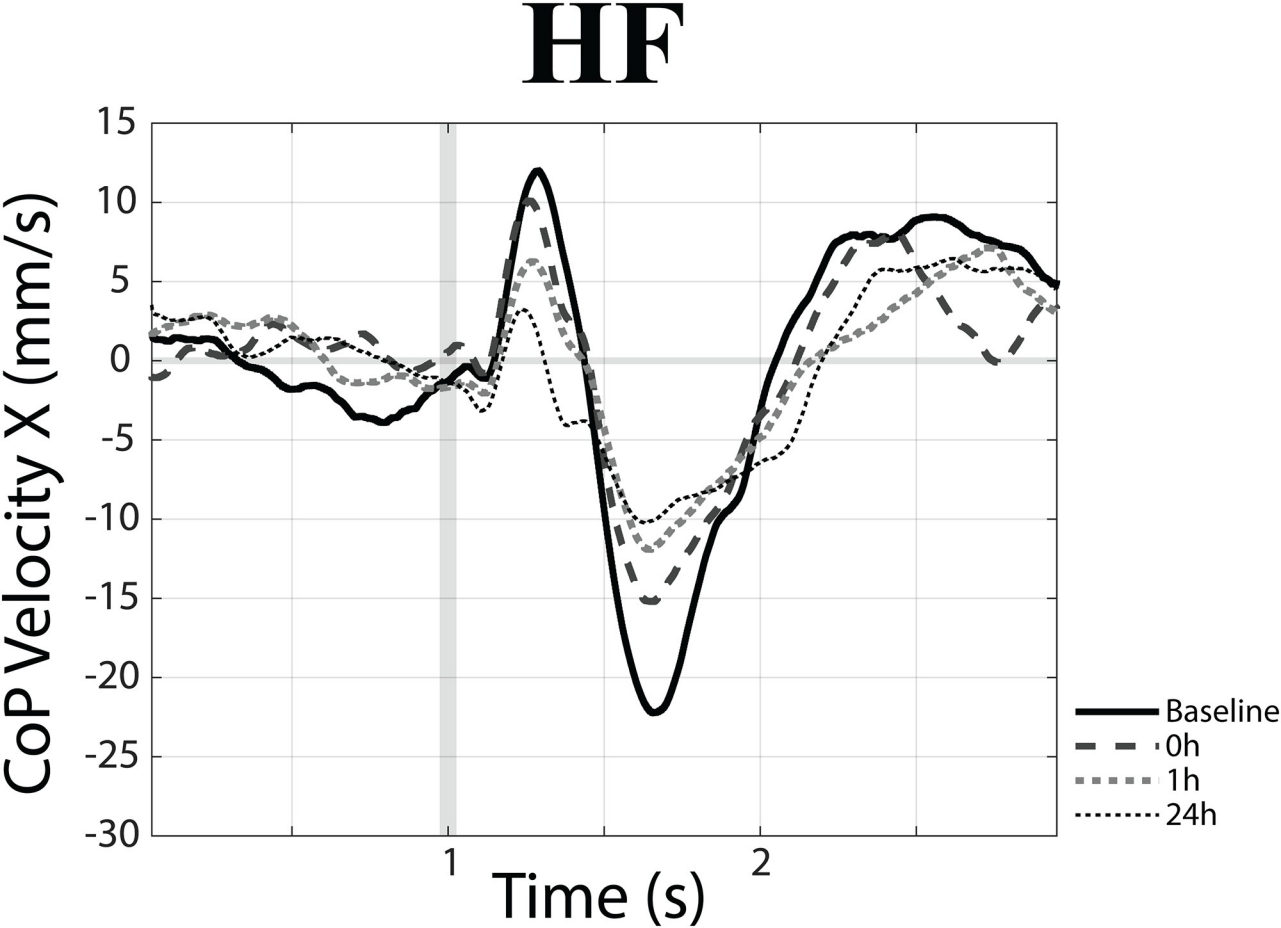
EVS-elicited changes in CoP velocity at different time points. EVS-elicited changes in the X component of CoP velocity observed in a representative subject (HF position) at different time points (baselinecontrol, 0h, 1h, 24h) with respect to postural training.

Within the training-EVS session a significant Time effect could be found for V (F(3,33) = 6.85, p<0.001) along the X axis. This effect was related to a decrease in V amplitude following postural training, illustrated in Fig 7 for a representative case.

**Fig 8:**
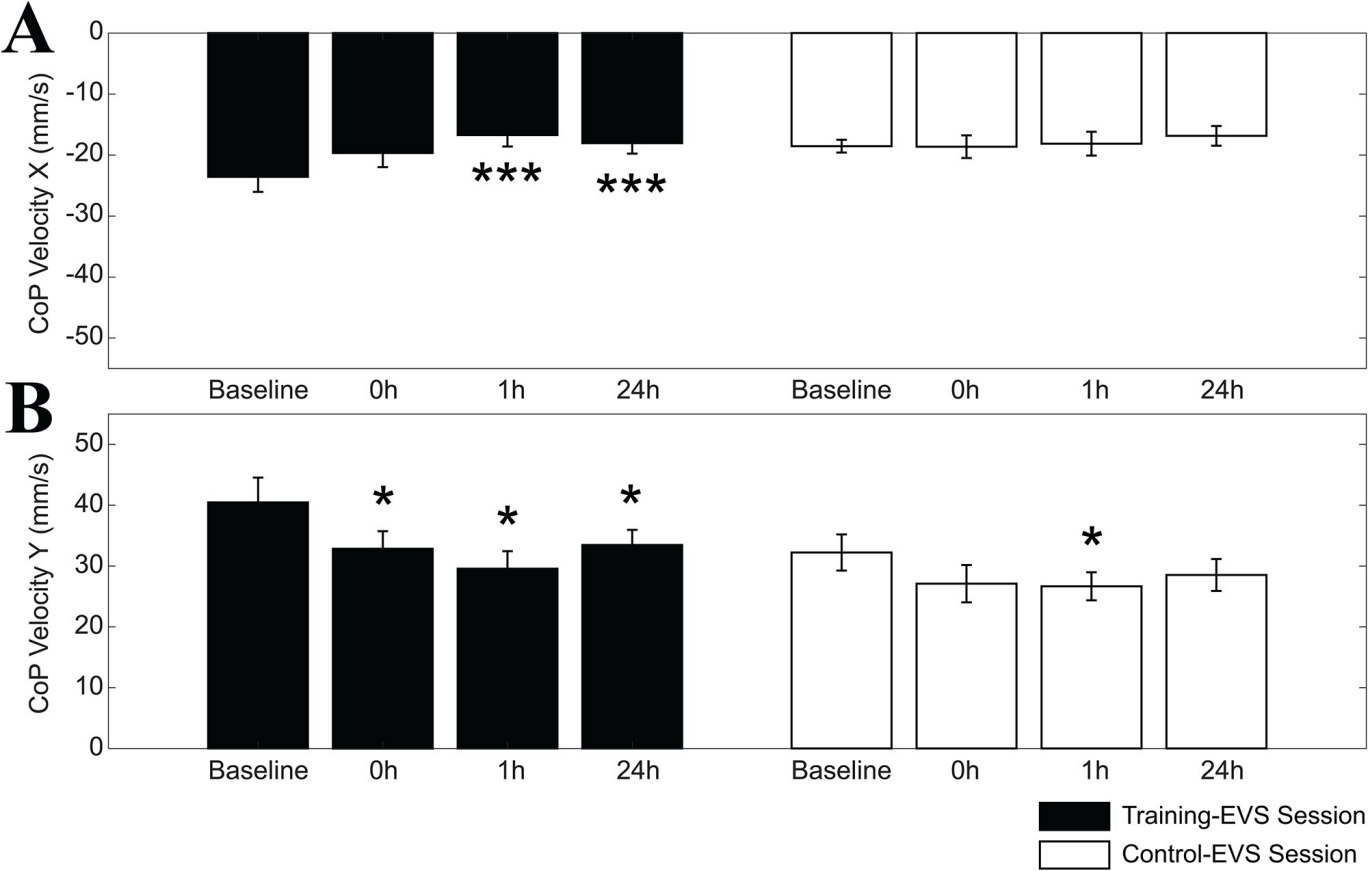
Time changes in X and Y components of CoP velocity to EVS. Mean±ES values of X (A) and Y (B) components of CoP velocity (V) changes evaluated at different time points. The training-EVS and the control-EVS sessions are represented by the black and white bars, respectively. Measurements were taken before the unipedal training/rest period (baseline), soon after (0h), as well as 1 hour (1h) and 24 hours (24h) later. Asterisks refer to significant differences with respect to baseline. *: p<0.05; **: p<0.02; ***: p<0.01.

As shown in Fig 8A, the V amplitudes (average of the values obtained for the different head positions) were significantly or quasi significantly reduced soon after (p = 0.064), at 1 hour (p = 0.007) and 24 hours (p = 0.01) following the adaptation period. As to the Y component of V, depicted in Fig 8B, a 4 Time x 2 Head position ANOVA revealed a significant Time effects for V (F(3,33) = 5.387, p = 0.028), with a significant reduction of the peak to peak excursion of CoP velocity soon after (p = 0.047), 1 hour (p = 0.029) and 24 hours (p = 0.023) following the adaptation period. No significant differences could be found for baseline values of both X and Y components between the control-EVS (X: 18.54±3.34, SD, mm/s; Y: 32.23±9.40, SD, mm/s) and the training-EVS (X: 23.56±8.54, SD, mm/s; Y: 39.28±13.55, SD, mm/s) sessions.

Qualitatively similar effects of training could be observed when the V1 and V2 components of the changes in CoP velocity were analysed separately: in this instance significant/quasi-significant Time effects could be observed along both the X (V1: F(3,33) = 3.014, p = 0.068, V2: F(3,33) = 8.211, p<0.0005) and Y (V1: F(3,33) = 3.741, p = 0.055; V2: F(3,33) = 4.991, p = 0.017) axes.

As to the Control-EVS session, no Time or Head x Time effect could be observed for V, V1 and V2 amplitudes along both axes. However, the Time effect for the Y component of V (F(3,27) = 2.694, p = 0.066) was not very far from the significance level. Moreover, post-hoc analysis indicated quasi-significant and significant differences of 0h (p = 0.058) and 1h (p = 0.024) time points with respect to the initial (baseline) value. These results are illustrated in Fig 8A/8B for X and Y velocity components, respectively. It must be pointed out that, when V amplitudes along the Y axis were expressed as differences versus the initial (baseline) values, no significant differences were found between the EVS-training and control session, whatever time point was considered. At variance, when the V amplitudes along the X axis were submitted to the same procedure, a significant difference was observed at 1h (p = 0.025) between the training-EVS and control-EVS session.

### Training-induced changes in postural stability and VSR

**Fig 9:**
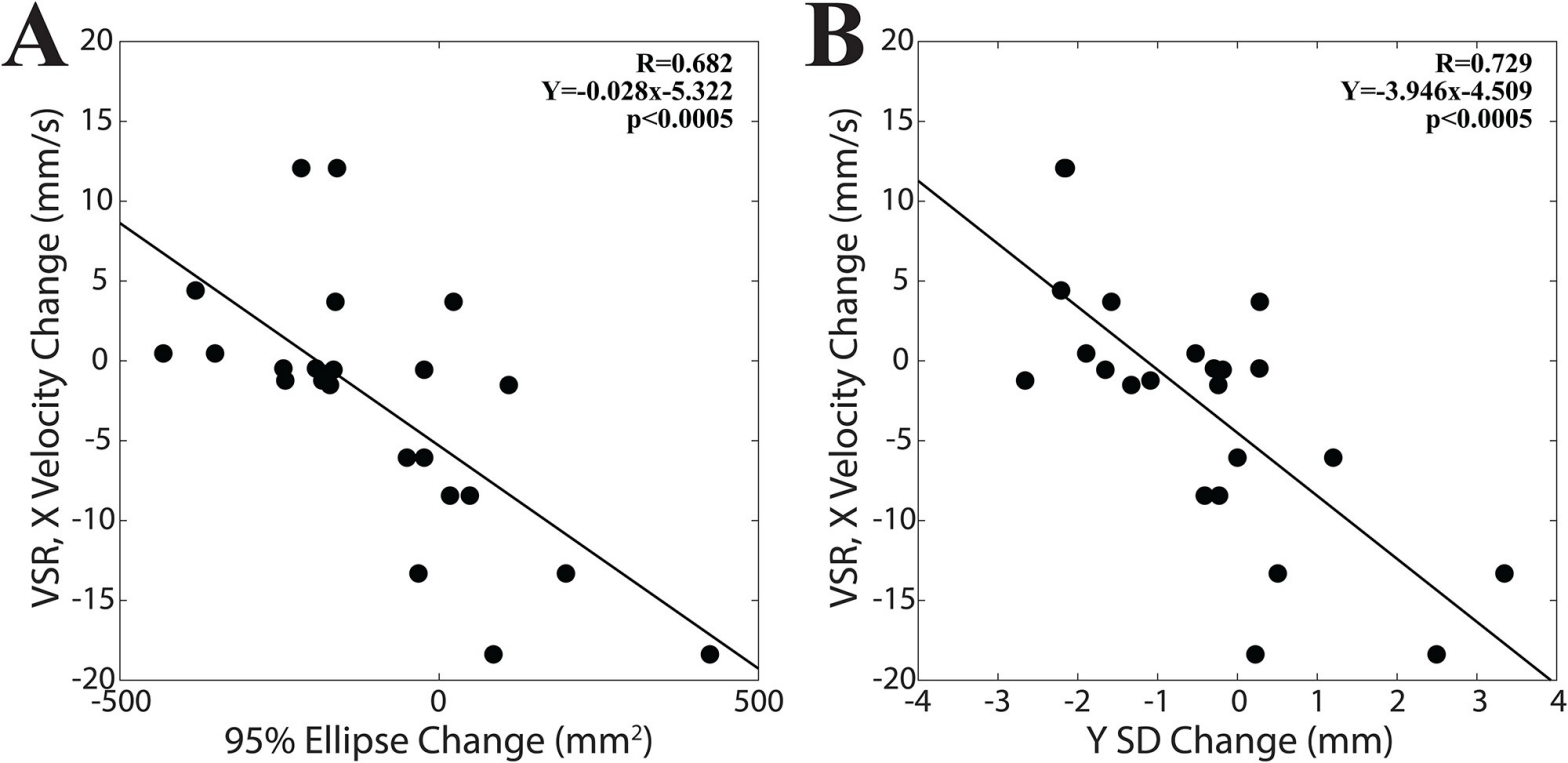
Correlations between changes in body sway and EVS-evoked CoP responses elicited by postural training. A. Relation between the changes observed during the last two time points of postural training in 95% ellipse (A) and Y SD (B), and those in the EVS-evoked CoP responses recorded along the X axis (HL position). In both A and B, negative values along the ordinate axis indicated largest drops of the peak-to-peak CoP velocity.

A regression analysis was performed between the changes elicited in unipedal stance parameters in the last 4.6 minutes (corresponding to the ninth and tenth time points) of postural training and the corresponding changes in CoP responses to EVS. For this analysis V values were taken as positive so that a negative difference in ordinate indicated a decrease relative to the baseline time point. Significant correlations were found only in the HL position. Fig 9 shows that, although postural training reduced VSRs and increased postural stability, subjects showing the largest drops in VSRs (negative values on ordinate) were those increasing less postural stability, expressed as a reduction in 95% ellipse, Y SD or CoP velocity (negative values on abscissas). Indeed, EVS-driven changes in CoP velocity along the X axis were negatively correlated with those in 95% ellipse (Fig 9A, R = 0.682, p<0.0005, Y = -0.028X–5.322), Y SD (Fig 9B, R = 0.729, p<0.0005, Y = -3.945X–4.509) and CoP velocity (not shown, R = 0.472, Y = -1.560X-7.818, p = 0.023).

As to the EVS-driven changes in Y CoP velocity, they were negatively correlated only with training-induced changes in X SD (R = 0.503, Y = -5.3774X–6.583, p = 0.014). These correlations indicated that subjects who decreased less their VSR were those decreasing more their postural oscillations.

### Training-induced changes in VSRs: EMG activity

**Fig 10:**
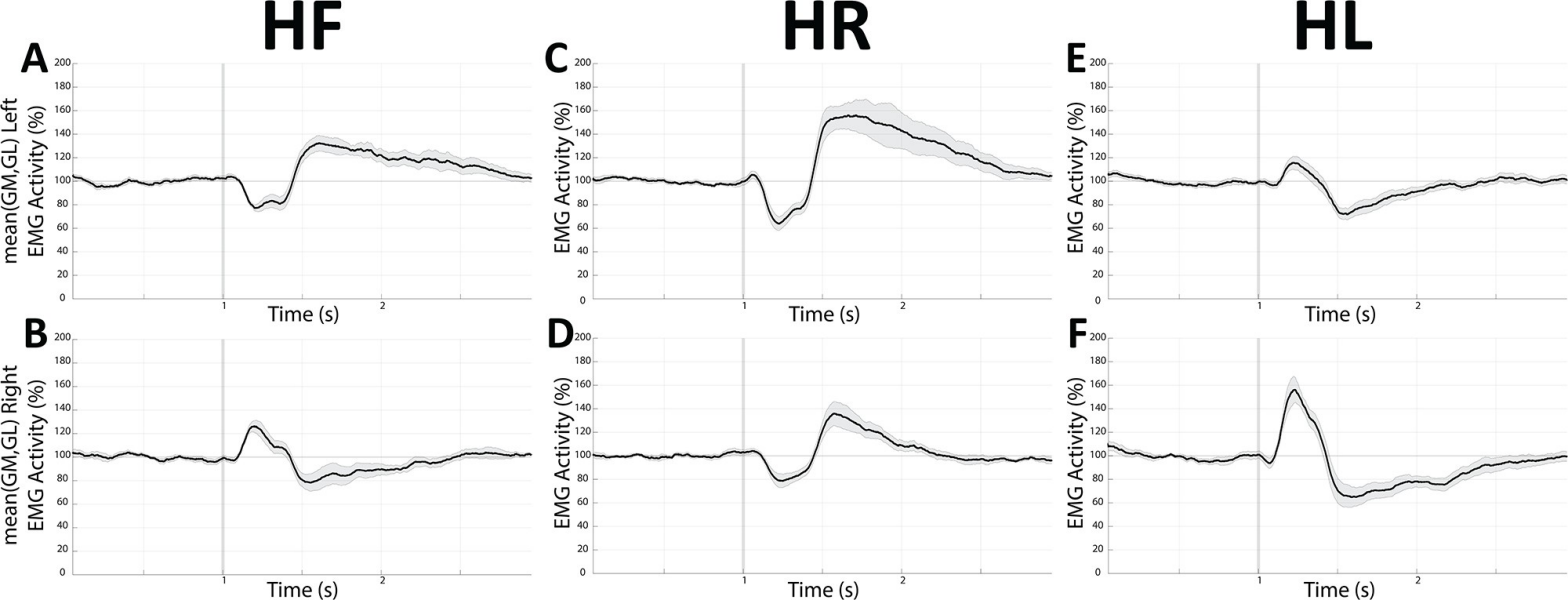
EVS-elicited changes in EMG activity. Grand average of the EMG response recorded from the left (A, C, E) and right (B, D, F) to EVS in HF (A, B), HR (C, D) and HL (E, F) position, at the beginning of the training session (baseline). All the subjects analysed over a 3 seconds time frame have been included (HF: n = 9, HL: n = 10, HR: n = 11). In each panel the grey area encompassing the average black line represents SE, while the vertical grey line is the stimulus onset. Data are expressed as a percentage of the average value evaluated over the pre-stimulus period.

EMG responses of plantar flexors locked to EVS are shown in Fig 10 for the left (A, C, E) and right (B, D, F) mean (GL and GM) activity. As expected, they depended upon the relative body-to-head position. In general, when the head was kept forward (HF), the EMG activity of both GL and GM was initially decreased on the left side (Fig 10A), while enhanced on the right side (Fig 10B). When the head was turned to the right side (HR), EMG responses consisted mostly in an initial bilateral depression of GL and GM (Fig 10C/10D) activity, larger on the left side. In the HL position, the EVS elicited on average an initial increase in the GM (Fig 10E/10F) and GL muscle activity, stronger on the right side.

**Fig 11:**
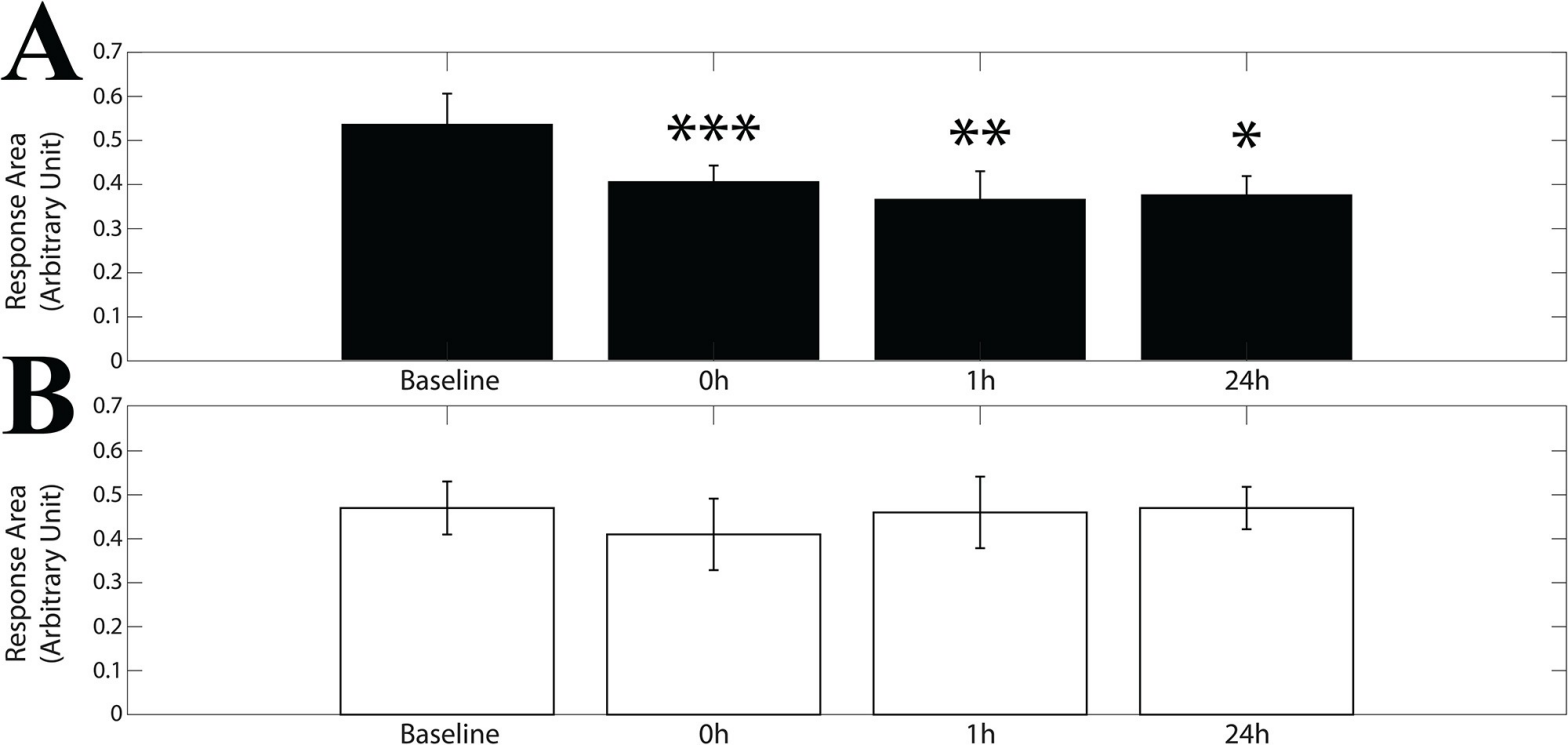
Training-related changes in EVS-evoked EMG responses of right and left muscles. Mean±SE of response area absolute values evaluated across different head positions (HF, HR and HL) and muscles (GM and GL) for both right (A) and left (B) sides. Measurements were taken before (baseline), soon after (0h), as well as 1 hour (1h) and 24 hours (24h) after the training/rest period. Asterisks refer to significant differences with respect to control. *: p<0.05; **: p<0.02; ***: p<0.01.

As shown in Fig 10 GM and GL data of the same side were averaged. Moreover, the response areas were all taken as positive and averaged across head positions. A 4 Time (baseline, 0h, 1h, 24h) repeated measure ANOVA applied to the averaged responses observed on the right side revealed a significant Time effect (F(3,30) = 4.885, p = 0.007), illustrated in Fig 11A. At variance, no significant time-related modifications could be observed on the left side responses (Fig 11B). Finally, these modifications were not dependent upon parallel changes in pre-stimulus EMG activity. Indeed, a 4 Time repeated measure ANOVA did not show significant Time effects for both left (F(3,24) = 1.454, p = 0.252) and right (F(3,30) = 0.910, p = 0.448) muscles (average of GL and GM across head positions), in spite of the significant changes observed in the EMG and CoP responses. Moreover, no correlation was found—at the level of individual muscles—between modifications in response area and in pre-stimulus activity (evaluated as a difference with respect to the baseline).

## Discussion

### A short-term form of postural learning improving unipedal stance

The present study, investigating four indicators of body sway (95% ellipse, CoP velocity and CoP oscillation along both X and Y axes), indicated that postural stability increased progressively in unipedal stance when this position was maintained 10 times for periods of 50 seconds spaced by sitting intervals of 90 seconds. Significant changes could be observed for all the parameters analysed, despite the limited sample size. The decreases in CoP velocity and in 95% ellipse were of similar amplitude so that the LFS values remained constant. These data show a more efficient control of body sway, which could be achieved through a change in the feedback mechanisms stabilizing posture [46–48]. Moreover, a modification could occur in the forward, centrally generated motor commands which are delivered on the basis of a body neural representation (forward model) [49]. There is indeed evidence that both feedback and forward mechanisms participate in the control of normal bipedal and unipedal stance [50, 51]. The large body oscillations occurring in unipedal stance are likely to increase the modulation of sensory signals converging at the level of central neural structures controlling posture [52–60]. Somatosensory and vestibular signals are particularly important in the challenging, unstable unipedal stance, since visual information are not appropriate for evoking fast, postural reaction, although it may enhance the response to the other inputs [61]. It is likely that the central convergence of posture-related signals (particularly of vestibular and somatosensory nature) may modify the excitability (and gain) of individual reflex pathways [62, 63] as well as the internal models [64] which sustain forward control mechanisms, leading to an improvement of body stability. Training-induced changes in postural variables were correlated to each other, except for CoP oscillation along X and Y axes: this finding is consistent with evidence indicating rather independent mechanisms [65] of vestibular control of postural stability along the two axes. The effects of training were stronger in those subjects showing the highest initial instability. This finding suggests that the learning process becomes less efficient when the postural stability is initially high, likely due to a decreased modulation of sensory afferents signalling body sway and to a reduced efficacy in changing the excitability of central pathways.

The effectiveness of postural training on postural stability was reduced by pre-training pseudo-random EVS, which continuously elicited VSRs. This procedure injected into the brain a labyrinthine input in the absence of a corresponding head movement, inducing a mismatch between somatosensory and vestibular signals and altering the central integration of vestibular and proprioceptive signals during unipedal stance. Such a mismatch may lead to a down regulation of the central processing of vestibular input which respect to the other sensory channels [66]. It is possible that this down regulation may last for few minutes, covering, at least in part, the following period of unipedal stance training and attenuating the changes in networks excitability which enhance stability during postural training.

EVS performed in HR and HL positions elicits simultaneous activation of balance reaction in the frontal and sagittal plane and this may increase the coupling between the corresponding neural control systems. This may explain why the changes elicited in postural sway along the X (X SD) and Y (Y SD) axes, although weaker with respect to those elicited in the training-stance session, are strongly correlated to each other.

### From unipedal to bipedal stance

No appreciable changes in postural parameters recorded in bipedal stance could be observed soon and 1 hour after the postural training performed in the more challenging unipedal stance. It has to be acknowledged, however, that the small sampling size might have hampered the detection of the less sizeable changes in postural parameters. At 24 hours from the training session a decrease in 95% ellipse and Y SD could be observed on soft support or with the eye closed. This observation is in agreement with studies showing that long-term postural learning may also modify normal stance condition in young athletes [13]. In the present report generalization of learning to bipedal stance took 24 hours to be observed. This time window encompassed a night of sleep. It is therefore possible that the beneficial effect of sleep documented for motor learning processes affecting voluntary movements [67, 68] may also extend to postural control mechanisms. A specifically designed sleep versus control study could test this hypothesis [69].

### Persistence of postural learning and translational applications

A limit of the present study was that the ability to maintain a challenging posture such as the unipedal stance was investigated only soon after the training session. It has been shown that the effects of a single session in postural training may persist the whole day, so that they may sum to those of another training session performed the day after [70]. This is probably the reason why repeated session of postural training regularly spaced within the week may lead in the long term to substantial improvements of postural stability [1, 5–14]. Such an improvement is not only exploited for improving postural control in sports performance, but also for rehabilitative purposes. Indeed, Vestibular rehabilitation programs (VRPs) use the plasticity of the balance system to speed up the natural compensation process that normally occurs following vestibular pathologies and are effective in enhancing gaze and postural stability, reducing vertigo, and improving daily living activities [71]. The training in unipedal stance that we have described could be adapted for VRP, which usually includes at least two daily sessions of increasing duration [72].

### Networks involved in the plasticity of postural functions

The training-induced changes in postural control may depend on plasticity mechanisms affecting several brain motor structures, which underlines general motor learning [73–75]. In this respect, it has been shown that postural training may lead to rapid and specific cortical grey matter changes [76] and modulates cortical excitability [30, 36, 41]. Another good candidate for contributing to postural learning is the cerebellum, which plays an important role in motor learning phenomena, such as visuomotor adaptation [77–79]. It has been shown that Purkinje cells undergo to significant changes in the density of their dendritic spine following an acrobatic training [80]. Indeed, the medial cerebellar vermal area controls stance [81–86] and promotes learning processes that modulates postural reflexes [63, 87–90], enhancing postural stability. Plastic changes of postural reflexes may be also elicited by manipulating the noradrenergic input impinging on the cerebellar vermis [91, 92], which arises from the Locus Coeruleus [93, 94].

### Postural learning and VSRs

A reduction in the velocity of CoP displacement elicited by EVS along the X and Y axes was clearly observed following postural training. A similar, less significant trend was observed for the Y component which, however, underwent some minor time-dependent attenuation also when the subject was not involved in the training sequence. This finding could be attributed to adaptation processes [66, 95], not observed for the X component of the vestibular response. This observation further emphasises the differences between neural mechanisms controlling postural stability on the sagittal and the frontal plane. The training-induced changes in CoP velocity could be attributed to the decrease of the EVS-evoked EMG responses observed at the level of the right GM and GL. In the present experiment, the stimulation was applied to the right side. Since the lateral vestibulospinal tract, which controls lumbar motoneurons, projects to the ipsilateral side of the spinal cord, this could explain the lack of changes on the contralateral muscle activation. However, we cannot exclude the hypothesis that contralateral changes have been undetected, due to the limited sample size. In addition, VSRs also involve neck, trunk and feet muscles [96–98] that were not investigated in the present study and could have contributed to the kinematic changes.

The depression of VSRs observed in the present investigation reminds the down regulation of H-reflex most often elicited by balance training [40, 99], via enhanced presynaptic inhibitory mechanisms [24, 26, 100, 101]. The latter phenomenon would be appropriate for increasing postural stability, which requires complex actions at multiple body levels [102], while the myotatic reflex has a more local extent of action. Moreover, delayed feedback responses associated with the myotatic reflex may lead to subsequent joint oscillations [25]. However, the vestibular system exerts a diffuse influence on body muscles [96–98] and the responses observed in the present study act in a feedforward manner [103], being developed before the sensory-based correction. In addition, although in the present experiment the VSR was depressed by postural training, which increased postural stability, unexpectedly subjects who decreased less the VSR were those decreasing more their postural oscillations (Fig 9). We may propose that postural training in unipedal stance acts by reducing the central, feedforward component of the postural oscillations, leading to a general weakening of postural reflexes, which are of reduced amplitude because of a stricter central control of CoP displacement. It is likely that the cerebellar structures involved in postural control play a key role in this process.
